# Neoadjuvant metformin added to conventional chemotherapy synergizes anti-proliferative effects in ovarian cancer

**DOI:** 10.1186/s13048-020-00703-x

**Published:** 2020-08-21

**Authors:** Kuo-Chang Wen, Pi-Lin Sung, Alexander T. H. Wu, Ping-Chieh Chou, Jun-Hung Lin, Chi-Ying F. Huang, Sai-Ching J. Yeung, Mong-Hong Lee

**Affiliations:** 1 Department of Obstetrics and Gynecology, Shuang Ho Hospital, Taipei Medical University, New Taipei City, 23561 Taiwan; 2 Department of Obstetrics and Gynecology, School of Medicine, College of Medicine, Taipei Medical University, Taipei, 11031 Taiwan; 3 Department of Obstetrics and Gynecology, Huei-Sheng Clinic, New Taipei City, 23561 Taiwan; 4 The Ph.D. Program for Translational Medicine, Taipei Medical University, Taipei, 11031 Taiwan; 5 Department of Molecular and Cellular Oncology, Division of Basic Science Research, The University of Texas MD Anderson Cancer Center, Houston, TX 77030 USA; 6 The University of Texas Graduate School of Biomedical Sciences, Houston, TX 77030 USA; 7 Department of Obstetrics and Gynecology, Taipei Veterans General Hospital and School of Medicine, National Yang-Ming University, Taipei, 11221 Taiwan; 8 Institute of Biopharmaceutical Sciences, National Yang-Ming University, Taipei, 11221 Taiwan; 9 Department of Emergency Medicine, Division of Internal Medicine, The University of Texas, MD Anderson Cancer Center, Houston, TX 77030 USA; 10 Guangdong Research Institute of Gastroenterology, The Sixth Affiliated Hospital of Sun Yat-sen University, 26 Yuancun Erheng Rd, Guangzhou, 510655 P.R. China

**Keywords:** Neoadjuvant metformin, Ovarian cancer, Clinically relevant dosage, AKT/mTOR pathway, Synergistic effects

## Abstract

**Background:**

Ovarian cancer is the leading cause of cancer-related death among women. Complete cytoreductive surgery followed by platinum-taxene chemotherapy has been the gold standard for a long time. Various compounds have been assessed in an attempt to combine them with conventional chemotherapy to improve survival rates or even overcome chemoresistance. Many studies have shown that an antidiabetic drug, metformin, has cytotoxic activity in different cancer models. However, the synergism of metformin as a neoadjuvant formula plus chemotherapy in clinical trials and basic studies remains unclear for ovarian cancer.

**Methods:**

We applied two clinical databases to survey metformin use and ovarian cancer survival rate. The Cancer Genome Atlas dataset, an L1000 microarray with Gene Set Enrichment Analysis (GSEA) analysis, Western blot analysis and an animal model were used to study the activity of the AKT/mTOR pathway in response to the synergistic effects of neoadjuvant metformin combined with chemotherapy.

**Results:**

We found that ovarian cancer patients treated with metformin had significantly longer overall survival than patients treated without metformin. The protein profile induced by low- concentration metformin in ovarian cancer predominantly involved the AKT/mTOR pathway. In combination with chemotherapy, the neoadjuvant metformin protocol showed beneficial synergistic effects in vitro and in vivo.

**Conclusions:**

This study shows that neoadjuvant metformin at clinically relevant dosages is efficacious in treating ovarian cancer, and the results can be used to guide clinical trials.

## Background

Ovarian cancer is the fifth leading cause of mortality in developed countries [1]. In the United States, an estimated 22,240 women were diagnosed with ovarian cancer in 2018, and 14,070 deaths due to ovarian cancer occurred [2]. Complete cytoreductive surgery followed by standard first-line platinum-taxene chemotherapy has been shown to improve the survival rate. However, the majority of patients experience relapse, and the 5-year survival rate is approximately 45% [3]. Chemoresistance to platinum-based treatment remains a major challenge in the successful treatment of ovarian cancer [4], and the mechanisms underlying platinum resistance are multifactorial. Various cellular processes are observed in resistant cells, and activation of the PI3K/AKT pathway is believed to be a determinant of resistance in ovarian cancer [5, 6]. Thus, the development of an improved treatment to overcome acquired resistance in cancer cells or decrease the side effects of platinum-based treatment is needed to treat ovarian cancer.

Metformin (N0,N0-dimethylbiguanide), a biguanide, is an oral hypoglycemia agent that is widely used as an antidiabetic drug to treat type 2 diabetes mellitus (DM); it is also widely used to treat polycystic ovarian syndrome [7]. Metformin has been shown to reduce cancer development in type 2 DM patients and inhibit growth in several cancer models [8, 9] either alone or in combination with cytotoxic agents [10, 11]. The major target of metformin in cancer cells is the tumor suppressor LKB1/AMP-activated protein kinase (AMPK) pathway, which serves as a metabolic checkpoint to arrest cell growth when intracellular ATP levels are low, such as in nutrient-poor conditions [12]. After activating AMPK, metformin phosphorylates tuberous sclerosis complex 2 (TSC2) and then binds with its obligate partner TSC1. TSC2 leads to the accumulation of Rheb–GDP and the inhibition of mTORC1, which influence eukaryotic translation initiation factor 4e-binding protein 1 (4eBP1) and ribosomal S6 kinase (S6K1), respectively. Shank et al. [13] showed that metformin can restrict the growth and proliferation of ovarian cancer stem cells. Yasmeen et al. [14] revealed that metformin induces apoptosis in ovarian cancer cell lines in an AMPK-independent manner by activating caspases 3/7, downregulating Bcl-2 and Bcl-xL expression, and upregulating Bax and Bad expression, which results in cell cycle arrest in the S and G2/M phases. Rattan et al. [15] identified metformin as an antiproliferative therapeutic that can act through both AMPK-dependent and -independent pathways; via these pathways, metformin inhibited cell proliferation in both wild-type and AMPK null mouse embryo fibroblasts as well as in AMPK-silenced ovarian cancer cells. In addition, metformin has been shown to inhibit PI3K/AKT/mTOR signaling in lung cancer [16, 17], breast cancer [18], pancreatic cancer [19], and hepatic cancer [20].

However, most studies showing that metformin alleviates cancer have used higher doses in vitro than those used in diabetic patients [8]. These high concentrations may directly cause the death of tumor cells. In the present study, we tested a low concentration as the effective dose, which was a clinically relevant dose. The effects of low-concentration metformin on AKT/mTOR signaling in ovarian cancer remain unclear.

The aim of the present study was to examine the effects of a combination of metformin at clinically relevant dosages and chemotherapy on ovarian cancer via the AKT/mTOR pathway. We found that metformin reduced ovarian cancer death in two clinical datasets and predicted that the effect of metformin in ovarian cancer was mediated by the AKT/mTOR pathway using a bioinformatics model. Then, we demonstrated that the low concentration of metformin inhibited the growth of a mouse ovarian surface epithelial cell line (MOSEC) and that it had a synergistic effect in combination with chemotherapy via the AKT/mTOR pathway. Neoadjuvant application of metformin plus chemotherapy yielded beneficial synergistic effects both in vitro and in vivo. The results provide insight into the potential of neoadjuvant metformin to augment the efficacy of existing cancer therapeutics.

## Results

### The effect of metformin on survival in ovarian Cancer patients

**Fig. 1:**
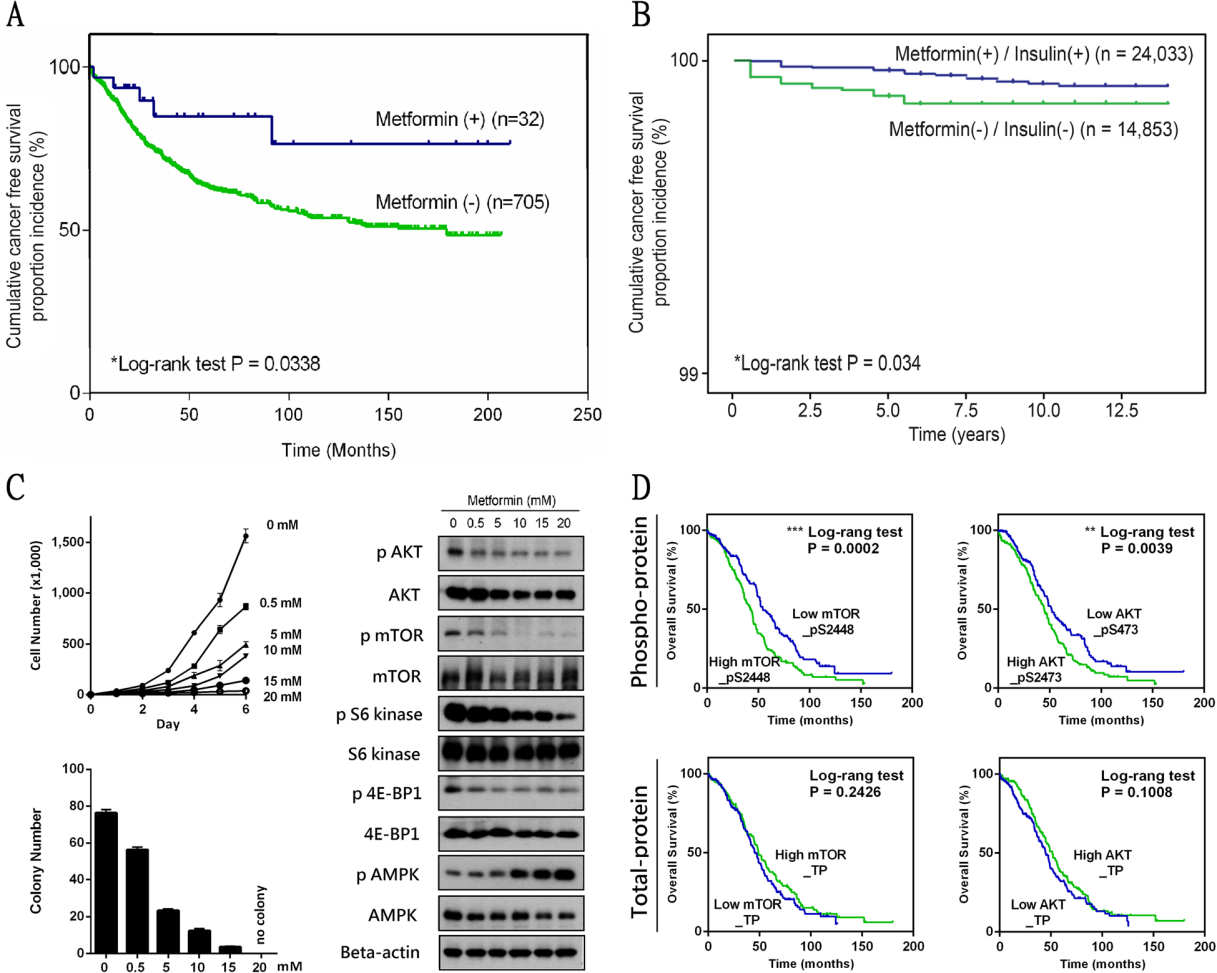
The Effect of Metformin on Survival in Ovarian Cancer Patients. **a** Kaplan-Meier OS of ovarian cancer patients with (*n* = 32) or without (*n* = 705) metformin use. **b** The ovarian cancer-free incidence in female DM patients: metformin(+) (ever used)/insulin(+) (ever used) users (*n* = 24,033) and metformin(−) (never used)/insulin(−) (never used) users (*n* = 14,853) from the National Health Insurance Taiwanese Dataset. **c** Tumor proliferation (cell number, 2D-colony formation) and Western blot analyses of the indicated proteins in the AKT/mTOR pathway of mouse ovarian cancer cells treated with different concentrations of metformin. **d** Comparison of the mortality rates between groups with low and high protein expression by the half-division approach. Kaplan-Meier analysis assessed the correlations of the indicated proteins (AKT [total and pSer473], mTOR [total and pSer2448]) with the overall survival of patients; data from the cBioPortal TCGA database (TCGA Provisional, ovarian cancer genomics, *n* = 606). A log-rank *p*-value less than 0.05 indicated a significant difference in overall prognosis (*p < 0.05, **p < 0.01, ***p < 0.001)

We investigated the impact of metformin on human ovarian cancer by analyzing a clinical dataset. In total, 797 patients were diagnosed with primary ovarian cancer in the Department of Gynecology and Obstetrics, Taipei Veteran General Hospital, from 1995 to 2012. After their clinical and drug histories were reviewed, 737 patients who underwent complete surgery and were treated with carboplatin were included in the analysis. Thirty-two of these patients took metformin either during admission or in the outpatient clinic. OS was measured from the date of diagnosis to death or was censored at the date of the last follow-up. The OS of patients with metformin treatment (*n* = 32) was significantly higher than that of patients without metformin (*n* = 705) (*p* = 0.03) (Fig. 1a, Table 1). Figure 1b shows the ovarian cancer-free incidence of female DM patients (*n* = 24,033 + 14,853) from the National Health Insurance Taiwanese Dataset. Ovarian cancer was less frequent among metformin(+)/insulin(+) users (*n* = 24,033) than among metformin(−)/insulin(−) users (*n* = 14,853) (*p* = 0.034*).* The use of metformin or insulin may help prevent ovarian cancer in female DM patients. We next investigated the cellular effects of metformin on ovarian cancer. The expression profiles of differentially expressed genes in response to treatment with metformin, chemotherapy or both were retrieved from the L1000 study. GSEA was performed using the up and down gene expression datasets from L1000. GSEA revealed that the gene expression induced by control and metformin treatment was similar to that of the KEGG pancreatic cancer pathway (http://www.genome.jp/dbget-bin/www_bget?pathway+hsa05212) (Fig. S1), which predominantly involved the AKT/mTOR pathway. Based on the proteomic and viability investigation, metformin may involve the alternation of phosphorylation in the AKT/mTOR pathway accompanying cell retardation in ovarian cancer, without affecting the total amount of protein (Fig. 1c). Metformin inhibited the AKT/mTOR pathway in a dose-dependent manner, as shown by Western blot analysis. The phenomenon was found at both high (15–20 mM), and low (0.5 mM) doses of metformin; low doses were closer to the clinically relevant concentration (Fig. S2). At low doses of metformin, the alternation of phospho-AMPK was not as obvious as that in the AKT/mTOR pathway. We further investigated the clinical role of the AKT/mTOR pathway using the TCGA ovarian cancer dataset. The patients with upregulated phosphor-protein, AKT_pSer473 or mTOR_pSer2448 had significantly poor OS; the expression of total protein was not associated with clinical importance in ovarian cancer (Fig. 1d). These findings highlight the value of metformin in inhibiting ovarian tumor cells via phosphorylation of the AKT/mTOR pathway, which indeed plays an essential role in the prognosis of ovarian cancer.

**Table 1:** The clinical characteristics of the study population

Characteristic	No metformin use *n* = 705 (96%)	Metformin use *n* = 32 (4%)	*p* value
Diabetes rate^%^	19 (2.7%)	32 (100%)	< 0.001 ***
Stage^a%^			
Early (I ~ II)	339 (51.1%)	16 (59.3%)	0.3168
Late (III ~ IV)	324 (48.9%)	11 (40.7)	
Histology^b%^			
Epithelial	542 (81.1)	24 (77.4)	0.5958
Other types^c^	126 (18.9)	7 (22.6)	
CA-125^d#^	1250 ± 273	767 ± 565	0.4109
Death rate^%^	239 (33.9%)	5 (15.6%)	0.0045 **
Overall survival^e§^	57.3 ± 4.2	69.7 ± 22.5	0.0338 *

^a^FIGO stage: *International Federation of Gynecology and Obstetrics*, surgical staging of ovarian cancer; missing value not included in statistical test

^b^Missing value not included in statistical test

^c^including *primary peritoneal serous carcinoma* (PPSC)

^d^before surgery, mean ± s.d. (U/ml)

^e^overall survival months, mean ± s.d.

^%^calculated by C*hi*-*square test*

^#^calculated by Student’s t-test

^§^calculated by Kaplan-Meier Log-Rank test

### Metformin at a clinically relevant dosage inhibits ovarian Cancer growth through the AKT/mTOR pathway

**Fig. 2:**
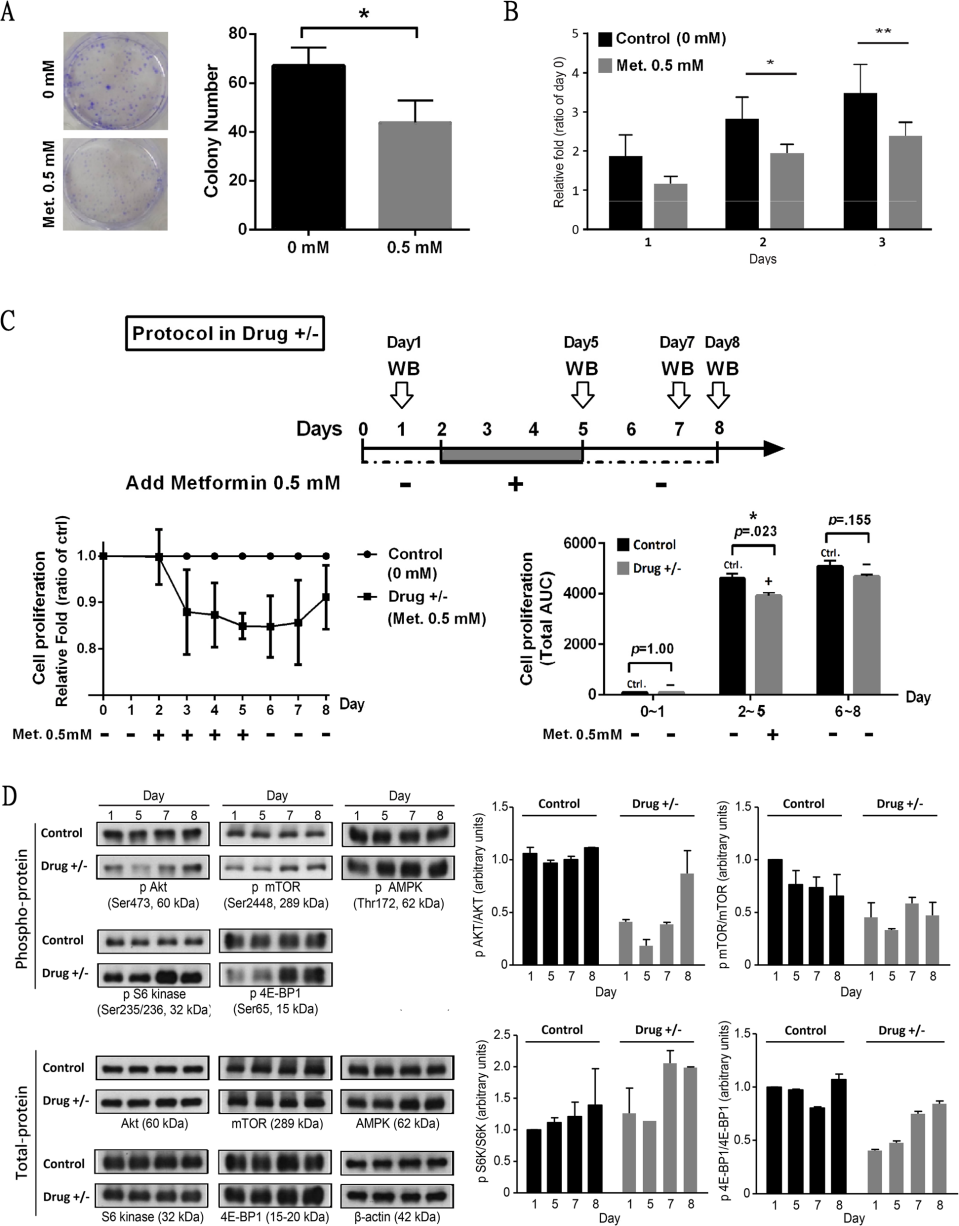
Metformin at a Clinically Relevant Dosage Inhibits Ovarian Cancer Growth through the AKT/mTOR Pathway. **a**-**b** Growth of mouse ovarian cancer cell line (MOSEC) in cells incubated for serial days with low-concentration metformin (0.5 mM). Met.: metformin. *: *p* < 0.05, by two-way ANOVA. **c** and **d** Cell viability and protein analyses of the AKT/mTOR pathway under treatment with metformin (0.5 mM) from day 2 to day 5 and when treatment was suspended from day 6 to day 8

To mimic the clinically relevant dosages in the human body, we used low-dose concentrations of metformin in the study. We assessed the cell growth of metformin in 0.5 mM-treated ovarian cancer cell lines to evaluate the growth-inhibitory effect of metformin. As shown in Fig. 2a-b, 0.5 mM metformin can reduce colony formation and cell growth: both mouse and human ovarian cancer cell lines manifested significantly reduced proliferation after treatment with clinically relevant doses of metformin (Fig. S3a). Furthermore, 0.5 mM metformin treatment beginning from day 2 to day 5 reduced cell viability, and cell viability recovered from day 6 to day 8 after discontinuing treatment (Fig. 2c). The inhibitory effect of low-dose metformin on the AKT/mTOR pathway was shown during metformin treatment (day 2 to day 5). The phospho-protein expression was inhibited during metformin treatment, but these expression levels were restored when suspending metformin (Fig. 2d). The aforementioned data suggested that metformin at low-dose concentrations could inhibit cell growth in ovarian cancer through inhibition of the AKT/mTOR pathway, as supported by the GSEA.

### The synergistic effects of metformin and chemotherapy on ovarian Cancer

**Fig. 3:**
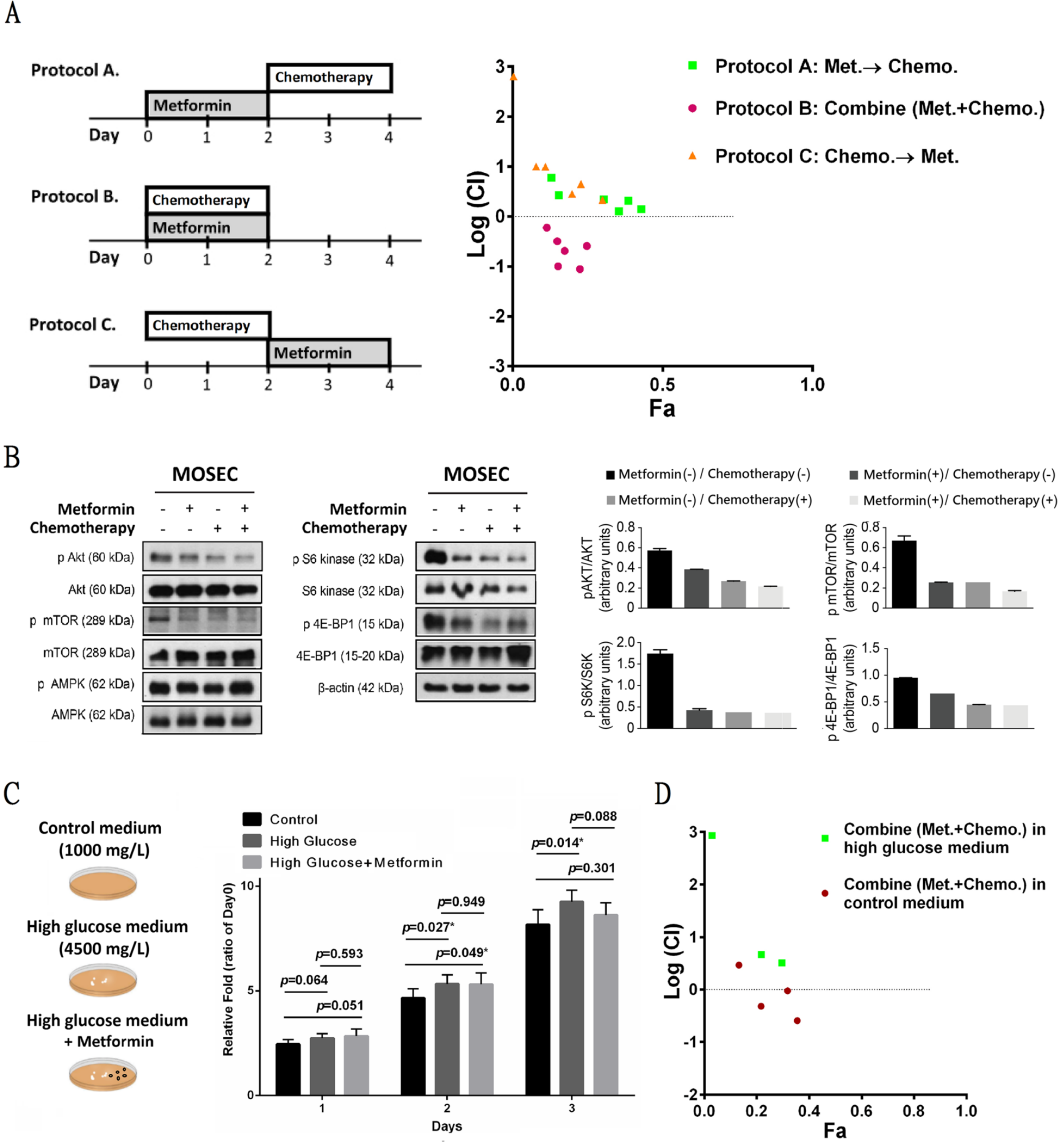
The Synergistic Effects of Metformin and Chemotherapy on Ovarian Cancer. **a** Synergistic effects of metformin combined with chemotherapy (carboplatin) at different concentrations and different combination protocols (concentration of metformin: 0.25 and 0.5 mM; concentration of carboplatin: 5, 10, and 50 μM). Met.: metformin; Chemo.: chemotherapy. **b** The effects of metformin alone (0.5 mM), chemotherapy (carboplatin, 50 μM) alone, or combined treatment assessed by the indicated antibodies in Western blot analysis. **c** MTT assays showed that cells cultured in a high-glucose medium (4500 mg/L) exhibited greater growth than those in control medium (1000 mg/L). Green squares represent cells cultured in high-glucose medium, and red solid circles represent those cultured in control medium (concentration of metformin: 0.25 and 0.5 mM; concentration of carboplatin: 10 and 50 μM).

Although standard first-line platinum-based protocols improve survival in ovarian cancer, strengthening these chemotherapy regimens is warranted. We evaluated the antiproliferative effects of different protocols (before, during, or after) and doses of metformin in combination with chemotherapy (Fig. 3a, left panel). As shown in Fig. 3a, concurrent combination of metformin and chemotherapy yielded the strongest inhibition. Forty-eight-hour exposure to concurrent metformin and chemotherapy resulted in a clear synergistic effect, with negative log (CI) values in ovarian cancer cells (Fig. 3a, right panel). Regarding human ovarian cancer cells, lower concentrations of carboplatin (5–50 μM) but not higher concentrations (100 μM) show synergy with metformin with negative log (CI) values (Fig. S3b). The activated forms of AKT are key intracellular mediators of growth, cell survival and platinum response. Determining the activation state of the AKT/mTOR pathway is important for understanding the synergistic mechanism of action of low-dose metformin combined with chemotherapy in ovarian cancer. Western blot analysis demonstrated that both chemotherapy alone and metformin alone reduced the levels of phosphorylated AKT/mTOR without affecting the total amount of AKT/mTOR protein (Fig. 3b). The combination of metformin and chemotherapy produced a stronger inhibition of pAKT and the AKT downstream effectors pmTOR (Ser2448), pS6 kinase (Ser235/236) and p4E-BP1 (Thr37/46) compared with metformin or chemotherapy alone. In contrast, the total amounts of mTOR, S6 kinase and 4E-BP1 were unaffected by treatment, and AMPK phosphorylation was not reduced by treatment with low-dose metformin or chemotherapy, either alone or in combination.

Some DM patients treated with metformin cannot achieve good blood sugar control and require other medications. We investigated the cellular effects of metformin treatment on tumors in patients with poor blood sugar control (Fig. 3c, left panel). As shown in Fig. 3c (middle panel), MTT assays indicated that cells cultured in high-glucose medium (4500 mg/L) showed accelerated cell proliferation relative to that of cells in control medium (1000 mg/L); however, metformin treatment diminished this effect induced by high glucose. The combined effect of metformin and chemotherapy in a high-glucose medium was antagonistic, with a positive log (CI) (Fig. 3c, right panel). To determine whether high glucose induces the AKT/mTOR pathway in MOSECs, we treated the cells with metformin alone, chemotherapy alone or a combination in high-glucose medium or control medium for 48 h (Fig. S4). High-glucose medium resulted in marked increases in pAKT, pmTOR, pS6 kinase and p4E-BP1 levels compared with the control medium. Treatment with metformin and carboplatin in the high-glucose medium reduced the protein level of pAKT but not the protein levels of pMTOR, pS6 kinase and p4E-BP1 relative to the levels in the control medium. These results revealed that high glucose or poor blood sugar control may diminish the antitumor effects of metformin and chemotherapy, although they still have effects on the pAKT/mTOR pathway.

### The beneficial synergistic effects of Neoadjuvant metformin under combination treatment

**Fig. 4:**
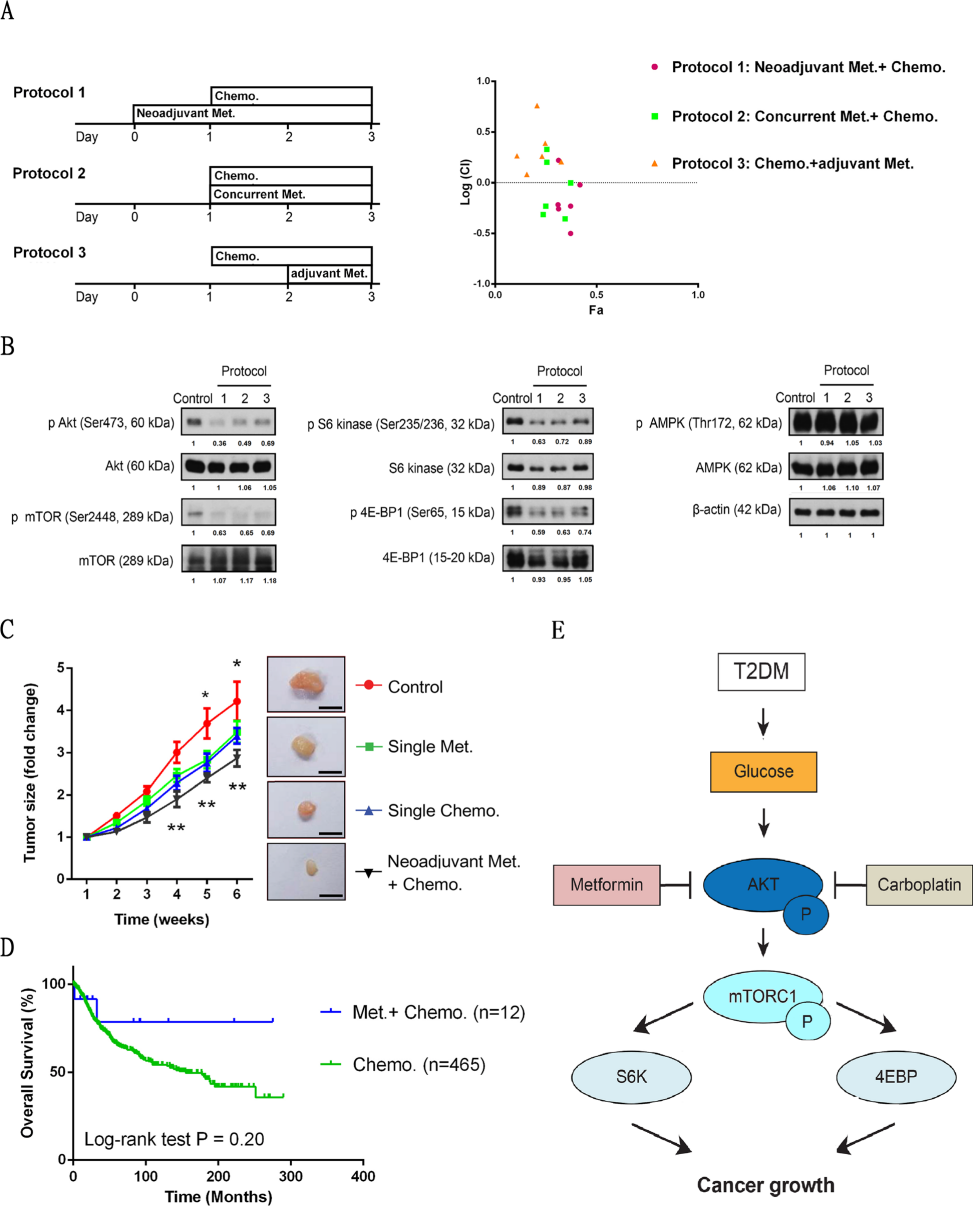
The Beneficial Synergistic Effects of Neoadjuvant Metformin under Combination Treatment. **a** and **b** Protocol 1: a neoadjuvant protocol, MOSECs treated with metformin alone for 1 day and then with a combination of metformin and carboplatin for 2 days. Protocol 2: a concurrent protocol, MOSECs treated with both metformin and carboplatin from day 2 for 2 days. Protocol 3: an adjuvant protocol, MOSECs treated with carboplatin alone from day 1 for 1 day and then with a combination of metformin and carboplatin for 1 day. Pink circles represent log (CI) values under protocol 1, green squares represent those under protocol 2, and yellow triangles represent those under protocol 3 (concentration of metformin: 0.25 and 0.5 mM; concentration of carboplatin: 5, 10 and 50 μM). Met.: metformin; Chemo.: chemotherapy. ImageJ analysis for relative intensity of protein bands. **c** MOSECs were injected into B6 mice (*n* = 20), which were divided into 4 groups, and subcutaneous tumor size was measured after different treatments (control, metformin or carboplatin alone, neoadjuvant metformin from Monday combined with carboplatin from Wednesday). Subcutaneous tumors were assessed at the end of the experiment. *: Control vs. Met.; *p* < 0.05. ** Control vs. Chemo., and Met. + Chemo.; *p* < 0.01. **d** Kaplan-Meier OS of ovarian cancer patients with (*n* = 12) or without (*n* = 465) metformin, in (before or during) chemotherapy. **e** The model of synergistic inhibitory effects by neoadjuvant metformin combined with chemotherapy in the AKT/mTOR pathway

We further investigated the synergistic effects of low concentrations of metformin and chemotherapy under different treatment regimens commonly used in clinical settings, as shown in Fig. 4a. Synergistic effects were obviously observed in both protocol 1 (neoadjuvant metformin) and protocol 2 (concurrent metformin) but not in protocol 3 (adjuvant metformin). Western blot analysis indicated that protocols 1–3 reduced the activities of pAKT, pmTOR, pS6 kinase and p4E-BP1, with protocols 1 and 2 having superior effects compared with protocol 3 (Fig. 4b). These results indicate that metformin should be used before or at least concurrent with chemotherapy to enhance the antitumor effect. Neoadjuvant metformin combined with chemotherapy was better than the combination protocol.

We further investigated the in vivo antitumor activity of neoadjuvant metformin in B6 mice bearing MOSECs that were grown subcutaneously as tumor xenografts. Treatment with metformin or chemotherapy as single agents caused a decrease in tumor size relative to that of control untreated mice. Treatment with the neoadjuvant metformin combined with chemotherapy significantly reduced tumor growth (Fig. 4c). Finally, we selected the cases with chemotherapy from Fig. 1a to determine the clinical impact of metformin in chemotherapy. Metformin before or during chemotherapy indeed showed a trend toward better overall survival compared with single chemotherapy, although this trend did *not reach statistical significance (*Fig. 4d).

Schematics of the intracellular effects of treatment with metformin, chemotherapy, or their combination on ovarian cancer are shown in Fig. 4e. Neoadjuvant metformin combined with chemotherapy produced better synergistic effects, inhibiting AKT and its downstream pathway. A high-glucose environment, such as that in poorly controlled type 2 DM patients, may increase activated AKT/mTOR signaling. Thus, for patients with poor glucose control, the combination of metformin and chemotherapy may be slightly superior to chemotherapy alone and not as efficacious as that in patients with good glucose control.

## Discussion

Diabetes is strongly associated with an increased incidence of cancer [21]. Many studies have shown that metformin can reduce the risk of cancer, including breast, colon, liver, and pancreatic cancers, and improve outcomes over those obtained with other antidiabetic treatments (sulfonylurea, insulin) in diabetic patients [9]. Whether metformin can reduce the risk of ovarian cancer has been investigated [22–24], but few studies have focused on the effects of metformin combined with commonly used first-line chemotherapeutic drugs, such as carboplatin, and the underlying mechanisms [25]. Neoadjuvant metformin combined with other therapies have been administered to treat ER-positive breast cancer in a Phase II clinical trial (ClinicalTrials.gov Identifier: NCT01589367). In this study, we evaluated the synergistic effects of neoadjuvant metformin combined with chemotherapy in ovarian cancer via the AKT/mTOR pathway both in vitro and in vivo and found that poor glucose control diminished the synergistic, antitumor effects of combination treatment.

A previous case-control study that used the UK-based General Practice Research Database [23] revealed that the adjusted odds ratio of metformin use vs. non-use for ovarian cancer incidence was not significant in non-diabetic patients but was significant in diabetic patients. In another recent meta-analysis, which included one observational study and two clinical trials, the pooled odds ratio (95% CI) of metformin use for ovarian cancer incidence was 0.67 (0.44–1.04) [26]. Recently, a study was performed using reimbursement databases of the National Health Insurance (NHI) to evaluate metformin use in Taiwanese women with type 2 DM. Metformin decreased the incidence of ovarian cancer, and the overall fully adjusted hazard ratio (95% CI) for ever-users versus never-users was 0.658 (0.593–0.730). In the present study, the results were similar to those of a previous report [24], in which ovarian cancer was less likely to occur in metformin(+)/insulin(+) users (*n* = 24,033) than in metformin(−)/insulin(−) users (*n* = 14,853). This previous study similarly used reimbursement databases of the NHI, although the data were from a different time period. The hospital cohort data showed that ovarian cancer patients treated with metformin had a significantly longer OS compared with patients not treated with metformin. This finding is similar to that of another case-control study, in which an association between metformin use and improved survival of ovarian cancer patients was identified [27]. These results indicated that metformin may reduce ovarian cancer incidence in type 2 DM patients and improve survival after a diagnosis of ovarian cancer.

Most studies examining the effects of metformin on cancer have used doses (1–10 mM) higher than those used clinically for diabetic patients [8, 28], yielding metformin plasma concentrations between 6 and 30 μmol/L. Few studies have focused on the impact of metformin at clinically relevant dosages [25]. Although Dr. Hu and colleagues administered low-dose metformin (0.1 mM or 0.01 mM) to suppress ovarian and breast cancer cell growth and survival in vitro, the aim of their study was to investigate whether low-dose metformin reprograms these cancer cells into noncancerous cells in a FOXO3-dependent manner, which may allow patients to successfully overcome these cancers with minimal side effects. However, they did not compare the inhibitory effects or therapy protocol of metformin combined with chemotherapy. In addition, they administered metformin via intravenous injection of metformin (5 mg/kg BW) in mice, which contrasts the more common oral route used in clinical applications, for example, diabetes mellitus [29]. In the present study, we tested a low concentration of 0.5 mM (500 μmol/L) as the effective dose. The cellular events observed in vitro suggest that this dose is safe and can be translated to in vivo conditions. The dosage of metformin given to mice was 150 mg/kg/day, which is equivalent to 720 mg/day for a 60-kg person according to a formula suggested by the National Institute of Health (U.S.A.) [30]. This equivalent dosage is 3 times lower than the maximum safe dosage of 2550 mg/day recommended in the Physician’s Desk Reference.

Metformin activates AMPK via LKB1, which leads to the inhibition of mTOR signaling and its major downstream effectors, the 4E-BPs and p70S6Ks, and the inhibition of global protein synthesis and proliferation in various cancer cell lines [6, 31]. In previous studies, metformin tested on ovarian cancer was found to induce cell cycle arrest, apoptosis [32], angiogenesis and decreased pmTOR expression [32], p38 MAPK pathway activity [33] and cancer stem cell activity [34]. In this study, we showed for the first time the effects of metformin on gene expression patterns using GESA and the L1000 system and found that the effects of metformin on gene expression in ovarian cancer are similar to those following activation of the KEGG pancreatic cancer pathway (Fig. S1). In previous studies, following metformin treatment, phospho-AKT levels decreased in two pancreatic cancer cell lines, A549 and PANC-1. We analyzed TCGA data and found that the phospho-AKT/mTOR pathway is a determinant of clinical survival in ovarian cancer [5, 6]. We also demonstrated, for the first time, the effects of metformin in combination with carboplatin, a first-line chemotherapy drug, and showed that the synergism of these drugs is due to the inhibition of the AKT/mTOR pathway, which is independent of AMPK at micromolar concentrations of metformin (0.5 mM). These results are consistent with those of Rattan et al. [15], who showed that metformin as an antiproliferative therapeutic can act through both AMPK-dependent and -independent pathways. Metformin can be a “very-very-very” inexpensive drug compared with AKT/mTOR inhibitors, and it may also have antitumor effects. Knowledge of this mechanism may be useful in clinical trials to adjust the dosage of chemotherapy or further overcome the chemoresistance to platinum in the future.

Metformin can be added at different times (before, during and after adjuvant chemotherapy), and the effects of these addition time points need to be investigated. In the present study, a synergistic effect (CI < 1) was observed in the neoadjuvant (protocol 1) and concurrent (protocol 2) protocols but not in protocol 3 (adjuvant). Neoadjuvant metformin (protocol 1) was better than the concurrent regimen. These results are similar to those of Erices et al. [25], although they did not assess the adjuvant use of metformin. The optimal time frame of neoadjuvant metformin before chemotherapy should be determined in future studies or clinical trials, although we observed an inhibitory effect of neoadjuvant metformin 1 day prior to chemotherapy in vitro and 2 days prior to chemotherapy in vivo. Furthermore, the ovarian cancer patients have a better prognosis with metformin combined with (before or during) chemotherapy compared with chemotherapy alone. Due to the small case number and retrospective analysis, our results are not significant. The optimal time frame for neoadjuvant metformin administration before chemotherapy will allow for the acquisition of the most ideal results in basic experiments and clinical analyses. These findings provide beneficial evidence that can guide the design of clinical trials. The differences among protocols observed in this study may be related to the cytotoxic effects of metformin on cancer stem cells [34], which can enhance the efficacy of neoadjuvant and concurrent chemotherapy by preventing the establishment of chemoresistant clones.

Some patients treated with metformin continue to show poor blood sugar control, and high blood glucose may diminish the antitumor effect of metformin. Karnevi et al. [19] reported that metformin can significantly reduce the proliferation of several pancreatic cancer cell lines under normal glucose conditions; however, they found that hyperglycemia reduced metformin-induced growth inhibition by enhancing the IGF-I response and activating AKT, which stimulated AMPK-Ser485 phosphorylation and impaired AMPK-Thr172. Zhuang et al. [35] obtained similar results in breast cancer and ovarian cancer cell lines, reporting that cancer cells became less responsive to metformin when glucose was increased to 10 mM. In a breast cancer cell line, under low-glucose conditions, metformin significantly decreased the phosphorylation of AKT and various targets of mTOR, whereas phospho-AMPK was not significantly altered. In the present study, we used an ovarian cancer cell line and demonstrated that a high-glucose medium decreased the response to metformin. The synergistic effects of carboplatin and metformin were abolished. The phosphorylation of AKT in low-glucose conditions (1000 mg/L) was substantially reduced by metformin, and phosphorylation levels of targets of mTOR (S6K and 4EBP1) were decreased relative to those in high-glucose conditions (25 mM). In ovarian cancer, phospho-AMPK was not significantly altered. The response to metformin was substantially altered in low-glucose conditions. Based on previous studies and our observations, we hypothesize that high glucose fuels glycolytic metabolism, which maintains cellular ATP levels when metformin blocks mitochondrial function. When glucose is limiting, cancer cells lack sufficient fuel to maintain glycolytic metabolism. Additionally, mTOR signaling is blocked in an AMPK-independent manner, enhancing metabolic deficiency. Cellular ATP is depleted, leading to energy collapse and cell death [34].

## Conclusions

In conclusion, low-concentration metformin treatment of patients with ovarian cancer may have antitumor effects and synergistic effects when used in combination with chemotherapy through the AKT/mTOR pathway. Neoadjuvant metformin is a more preferable protocol than the concurrent regimen. Future prospective clinical trials in patients with ovarian cancer are required to investigate the beneficial effects of neoadjuvant metformin in augmenting the efficacy of existing cancer therapeutics.

## Methods

### Patient samples from Taipei veteran general hospital medical center

A total of 797 patients were diagnosed with primary ovarian cancer in the Department of Gynecology and Obstetrics, Taipei Veteran General Hospital, from 1995 to 2012. After a review of the patients’ clinical and drug histories, 737 patients who underwent complete surgery and were treated with platinum-based therapy plus paclitaxel were included in the analysis. Of these patients, 32 were identified as having taken metformin, either during admission or in the outpatient clinic. The overall survival (OS) was measured from the date of diagnosis to death or was censored at the date of the last follow-up. All documents were collected under protocols approved by the institutional review board of the hospital.

### Patient samples from the National Health Insurance Taiwanese Dataset

The reimbursement data of Taiwanese female patients with a new diagnosis of type 2 DM between 2000 and 2010 (*n* = 38,886) were retrieved from the National Health Insurance database. Among these patients, none used only insulin or only metformin. Therefore, we compared two groups: (1) those who received metformin and insulin (*n* = 24,033) and (2) those who received neither metformin nor insulin (*n* = 14,853). Then, we followed the two groups for newly diagnosed ovarian cancer from 2000 to 2011. Thirty-seven patients across the two groups were diagnosed with ovarian cancer.

### Microarray analysis

The microarray experiments were conducted following the L1000 Operating Procedure (L1000 SOP) [36]. Briefly, the human ovarian cancer cell line ES-2 was left untreated (control) or treated with micromolar concentrations of metformin (0.5 mM), 50 μM carboplatin, or a combination in a microplate. After 6 h of drug treatment, the medium was removed, and lysis buffer was added (included in the L1000 kit) to the wells for 30 min. After cell lysis, the lysate was stored at − 80 °C for at least one night before being transferred to a 384-well plate, which was performed using the protocol available at http://s3.amazonaws.com/support.lincscloud.org/protocols/data_generation/L1000_SOP.pdf. Gene expression profiles were detected by L1000 array technology. Up and down probesets were selected by performing two-sample t-tests; genes with expression differences significant at a *p* value < 0.01 and with fold changes > 1.5-fold were included. The up and down probesets were input into GSEA software for analysis and to interpret the transcriptional profile data of the four groups by GSEA methods [37, 38].

### Analysis of ovarian cancer using the cancer genome atlas (TCGA) genomics data

Clinical data and protein expression data of ovarian cancer from TCGA were downloaded from the cBioPortal website (http://www.cbioportal.org/) [39, 40]. Patients in the ovarian cancer (cBioPortal TCGA, provisional, ovarian cancer genomics, *n* = 606) dataset were categorized into low and high protein expression groups by a half-division approach. These two groups of patients were input as “User-defined Case List” to assess the total and phospho-protein levels, as evaluated by the RPPA z-score, of ±0, including those of key proteins involved in the AKT/mTOR pathway and AMPK. Kaplan-Meier analyses were performed to assess the correlations among the indicated proteins (AKT [total and pSer473], mTOR [total and pSer2448], and AMPK [total and pThr172]).

### Cell lines, cell culture, chemicals, and antibodies

The MOSEC line was a kind gift from Dr. Honami Naora (The University of Texas MD Anderson Cancer Center). Stable MOSEC lines were generated as previously described [41]. The MOSEC lines were cultured in DMEM medium [42]. The human ovarian cancer cell line SKOV3 and ovarian clear cell carcinoma cell line ES-2 were provided by Dr. Gordon Mills (The University of Texas MD Anderson Cancer Center) and Dr. Patrice Morin (National Institute on Aging, Baltimore, Maryland, USA), respectively and were cultured in McCoy’s 5A medium. All cell culture reagents used were obtained from Invitrogen (Thermo Fisher Scientific Inc., Waltham, MA, USA). Metformin (Sigma-Aldrich, St. Louis, MO, USA) was dissolved in DMEM containing 10% fetal bovine serum (FBS) at the indicated concentration. Carboplatin (Sigma-Aldrich) was dissolved in water and diluted in DMEM to various concentrations. Primary antibodies against AMPKα, phospho-AMPKα (Thr172), AKT, phospho-AKT (Ser473), mTOR, phospho-mTOR (Ser2448) S6, phospho-S6 (Ser235–236), 4EBP1, phospho-4EBP1 (Thr37/46) and β-actin were obtained from Cell Signaling Technology (Danvers, MA, USA). All other chemicals were purchased from Sigma-Aldrich.

### In vitro cell viability assays and cell proliferation assay

To assay cell viability following 0.5 mM metformin treatment, we seeded MOSECs in 12-well plates (2 × 10^4^ per well), cultured the cells for 1 to 5 days in medium containing 0.2% FBS, and collected and stained the cells with trypan blue for quantification at different time points. Day 0 represents the day of treatment. Cell proliferation was measured with the MTT assay, 2D colony-formation assay [43], or the sulforhodamine B (SRB) assay [44]. Briefly, MOSECs were seeded in 96- or 6-well plates and treated with different concentrations of metformin, carboplatin or both for the indicated times. The results were analyzed as described by Chou [45] using the CompuSyn program downloaded from http://www.combosyn.com/. The IC_50_ values for each drug were determined by interpolation from the dose-response curves. The resulting combination index (CI) is a quantitative measure of the degree of interaction between different drugs. CI = 1 denotes additivity; CI > 1 denotes antagonism; and CI < 1 denotes synergism. For interpretation, the combination was plotted as the log10(CI) versus the fraction affected (Fa; defined as 1–survival fraction). On these plots, additivity was defined as log (CI) = 0, synergy was defined as log10(CI) < 0; and antagonism was defined as log10(CI) > 0. All of the results were experimentally reproducible.

### Western blot analysis

Cancer cells were treated with control vehicle, metformin, carboplatin, or a combination for 48 h, and the cells were pelleted by centrifugation and rinsed with PBS. The cell pellets were then lysed in RIPA buffer followed by sonication. Lowry assays (Bio-Rad) were performed to determine the protein concentration. Equal amounts of protein were loaded in each lane and resolved by 10 to 12% gradient Bis-Tris gels. All Western blot analyses were performed using whole-cell lysates prepared as described above. SDS-PAGE and Western blotting were performed using standard methods. The protein band intensities were quantified using ImageJ analysis by determining the relative intensity for each experimental band and normalizing its absolute intensity to that of the control.

### Tumor Xenografts in a mouse model

C57BL/6 (B6) mice (4 weeks of age) were purchased from Taiwan National Laboratory Animal Center and LASCO laboratory. The research protocol was approved, and the mice were maintained in accordance with the Institutional Guidelines of Taipei Medical Center and Taipei Veteran General Hospital. MOSECs (1 × 10^6^ cells) were subcutaneously injected into the right flank of B6 mice (6 weeks of age). One week post-injection, mice were randomly divided into 4 groups: a control group, an oral metformin (150 mg/kg once per day) group, an IP carboplatin (30 mg/kg twice a week: Wednesday and Friday) group, and a combined-treatment (neoadjuvant metformin from Monday, combined with carboplatin from Wednesday) group. There were 5 mice per group (20 total). Drugs were applied 1 week after tumor injection, which was designated week 0 in all groups. Tumor length and width were measured using a caliper, and tumor volume was calculated using the following formula: volume = [length×width^2^]/2. The change in tumor size is expressed as the fold change in tumor volume. The fold change in tumor size each week was calculated as follows: fold change in tumor size = (week)n/tumor size initial (week 1). At the end of the experiment, the mice were sacrificed, and tumor samples from each group were collected for Western blot analysis.

### Statistical analysis

Statistical analysis was carried out using the PASW package (PASW Statistics V18, Chicago, IL, USA). Survival analysis was based on the Kaplan-Meier method. Comparisons of clinical characteristics between two groups were performed by Student’s t-test, the chi-square test or Fisher’s exact test. Comparisons between survival curves were performed using the log-rank or Breslow test. Comparisons of relative fold-changes in tumor cell survival among different treatment groups were performed by 2-way ANOVA with Bonferroni post-tests. A value of *p* < 0.05 was considered statistically significant.

## Supplementary information

**Additional file 1: Figure S1.** KEGG pathway. KEGG pancreatic pathway from http://www.genome.jp/kegg-bin/show_pathway?hsa05212. The cellular effects of metformin in ovarian cancer based on GSEA of up and down gene expression from an L1000 dataset.

**Additional file 2: Figure S2.** The effect of different concentrations of metformin in ovarian cancer cells. Tumor proliferation (2D-colony formation) of mouse ovarian cancer cells treated with different concentrations of metformin. Maximum plasma concentrations (Cmax) of Metformin was clinically around 1.03–4.12 mg/L (https://www.accessdata.fda.gov/drugsatfda_docs/label/label/2008/020357s031,021202s016lbl.pdf).

**Additional file 3: Figure S3.** Metformin at a Clinically Relevant Dosage Inhibits Human Ovarian Cancer Growth. a Growth of human ovarian cancer cells (SKOV3) incubated f with micromolar concentrations of metformin (0.5 mM) for several days. *: *p* < 0.05, two-way ANOVA. b The log (CI) values following a 48-h exposure to combination treatment of metformin and carboplatin, reflecting the synergistic effects against the human ovarian cancer cell line ES-2.

**Additional file 4: Figure S4.** The effects of metformin in normal- or high-glucose culture medium. Western blotting was performed to assess the expression of AMPK, AKT, MTOR, 4E-BP1 and S6 following treatment with metformin, chemotherapy (carboplatin), or both for 48 h in the presence or absence of a high-glucose medium. Beta-actin was included as a loading control.

## Data Availability

The datasets used and/or analyzed during the current study are available from the corresponding author on reasonable request.

## Abbreviations

DM: Diabetes Mellitus
AMPK: AMP-activated Protein Kinase
TCGA: The Cancer Genome Atlas
OS: Overall Survival
GSEA: Gene Set Enrichment Analysis
MOSEC: Mouse Ovarian Surface Epithelial Cell Line
TSC2: Tuberous Sclerosis Complex 2
4eBP1: 4e-Binding Protein 1
S6K1: Ribosomal S6 Kinase
L1000: L1000 Operating Procedure
