# Supplementary figures and images for: Neoadjuvant metformin added to conventional chemotherapy synergizes anti-proliferative effects in ovarian cancer
Source: J Ovarian Res. 2020 Aug 21;13:95. doi: 10.1186/s13048-020-00703-x (PMC7442990; doi:10.1186/s13048-020-00703-x)

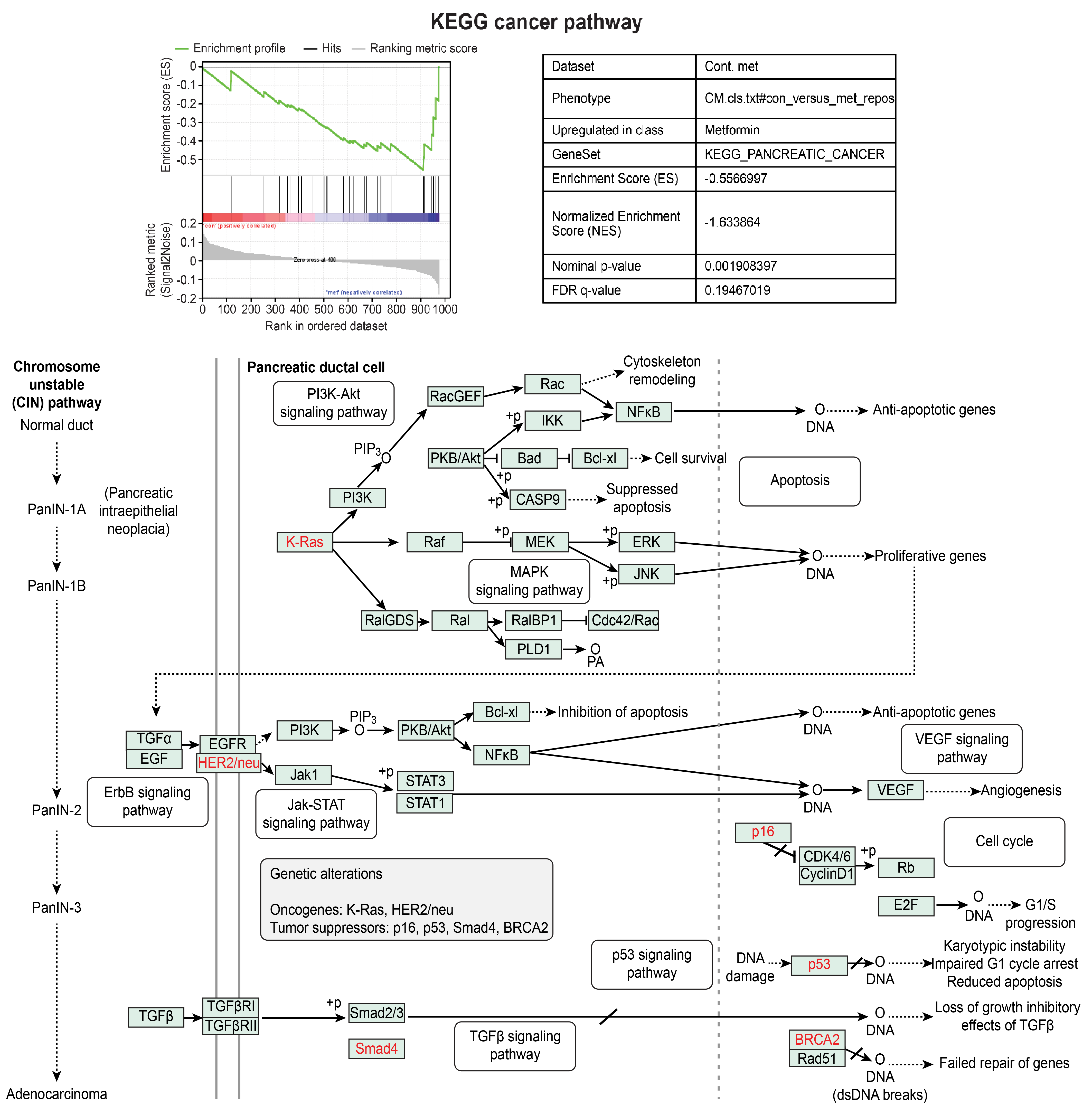

Supplement: Supplementary file 1 — Additional file 1: Figure S1. KEGG pathway. KEGG pancreatic pathway from http://www.genome.jp/kegg-bin/show_pathway?hsa05212. The cellular effects of metformin in ovarian cancer based on GSEA of up and down gene expression from an L1000 dataset. [file 13048_2020_703_MOESM1_ESM.tif]

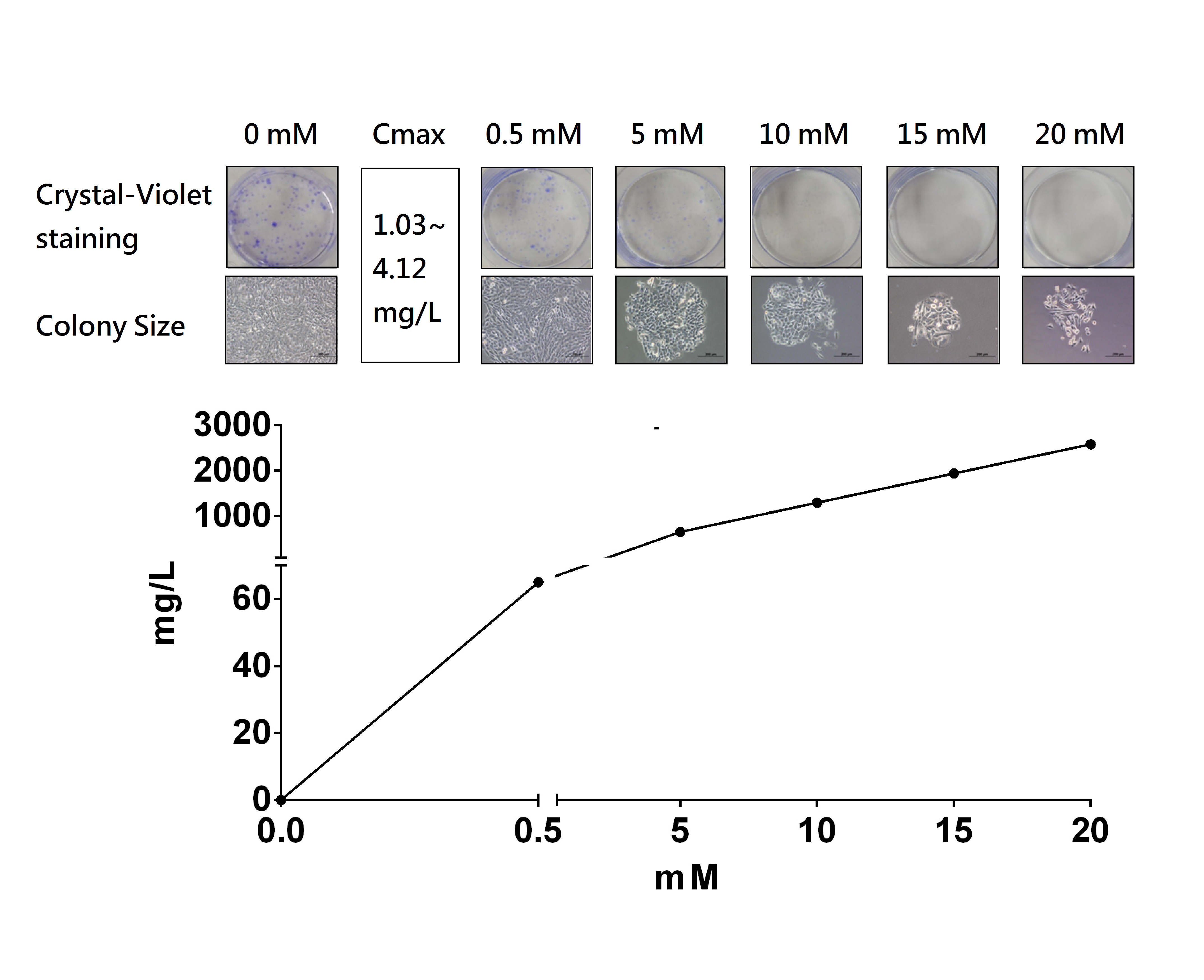

Supplement: Supplementary file 2 — Additional file 2: Figure S2. The effect of different concentrations of metformin in ovarian cancer cells. Tumor proliferation (2D-colony formation) of mouse ovarian cancer cells treated with different concentrations of metformin. Maximum plasma concentrations (Cmax) of Metformin was clinically around 1.03–4.12 mg/L (https://www.accessdata.fda.gov/drugsatfda_docs/label/label/2008/020357s031,021202s016lbl.pdf). [file 13048_2020_703_MOESM2_ESM.tif]

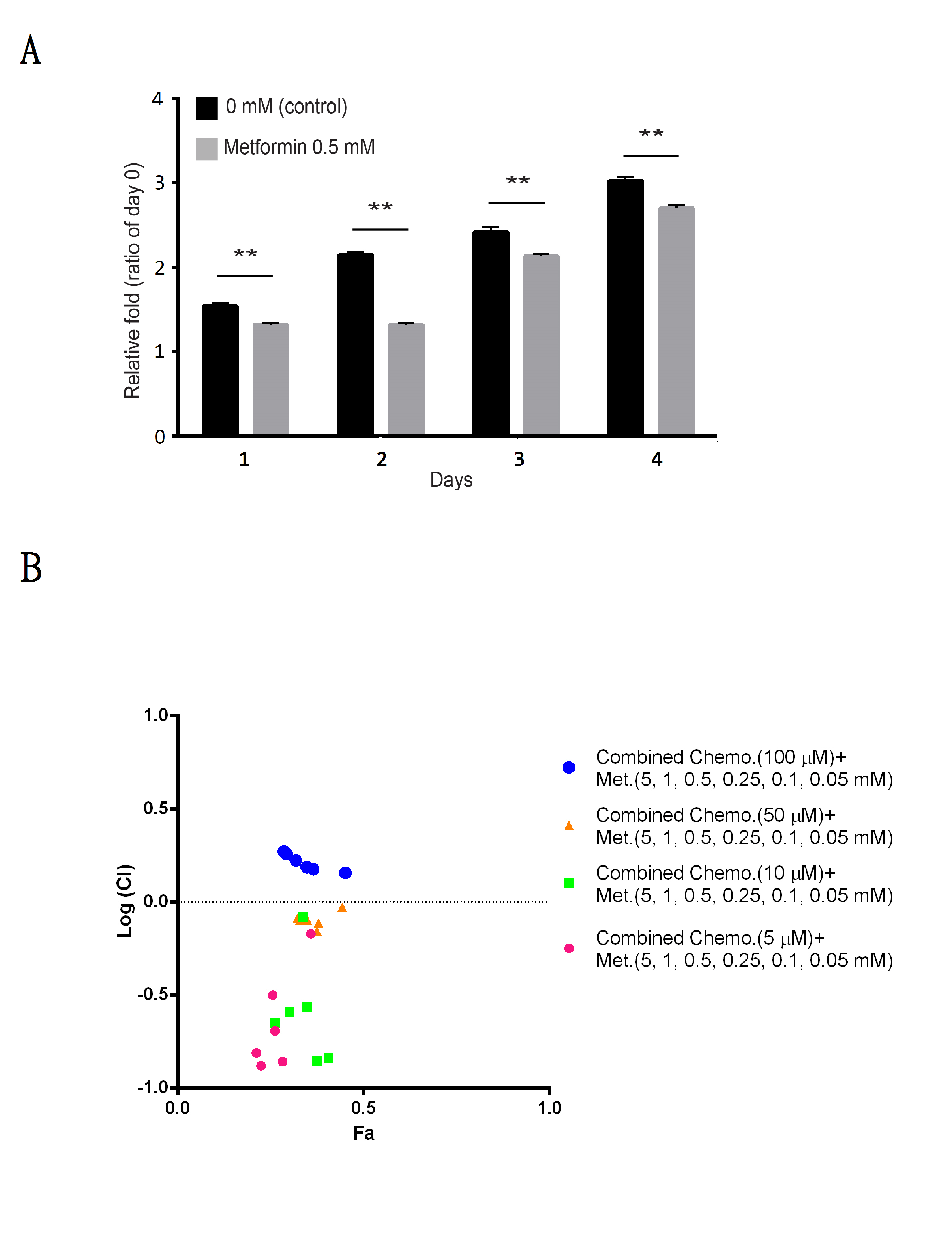

Supplement: Supplementary file 3 — Additional file 3: Figure S3. Metformin at a Clinically Relevant Dosage Inhibits Human Ovarian Cancer Growth. a Growth of human ovarian cancer cells (SKOV3) incubated f with micromolar concentrations of metformin (0.5 mM) for several days. *: p < 0.05, two-way ANOVA. b The log (CI) values following a 48-h exposure to combination treatment of metformin and carboplatin, reflecting the synergistic effects against the human ovarian cancer cell line ES-2. [file 13048_2020_703_MOESM3_ESM.tif]

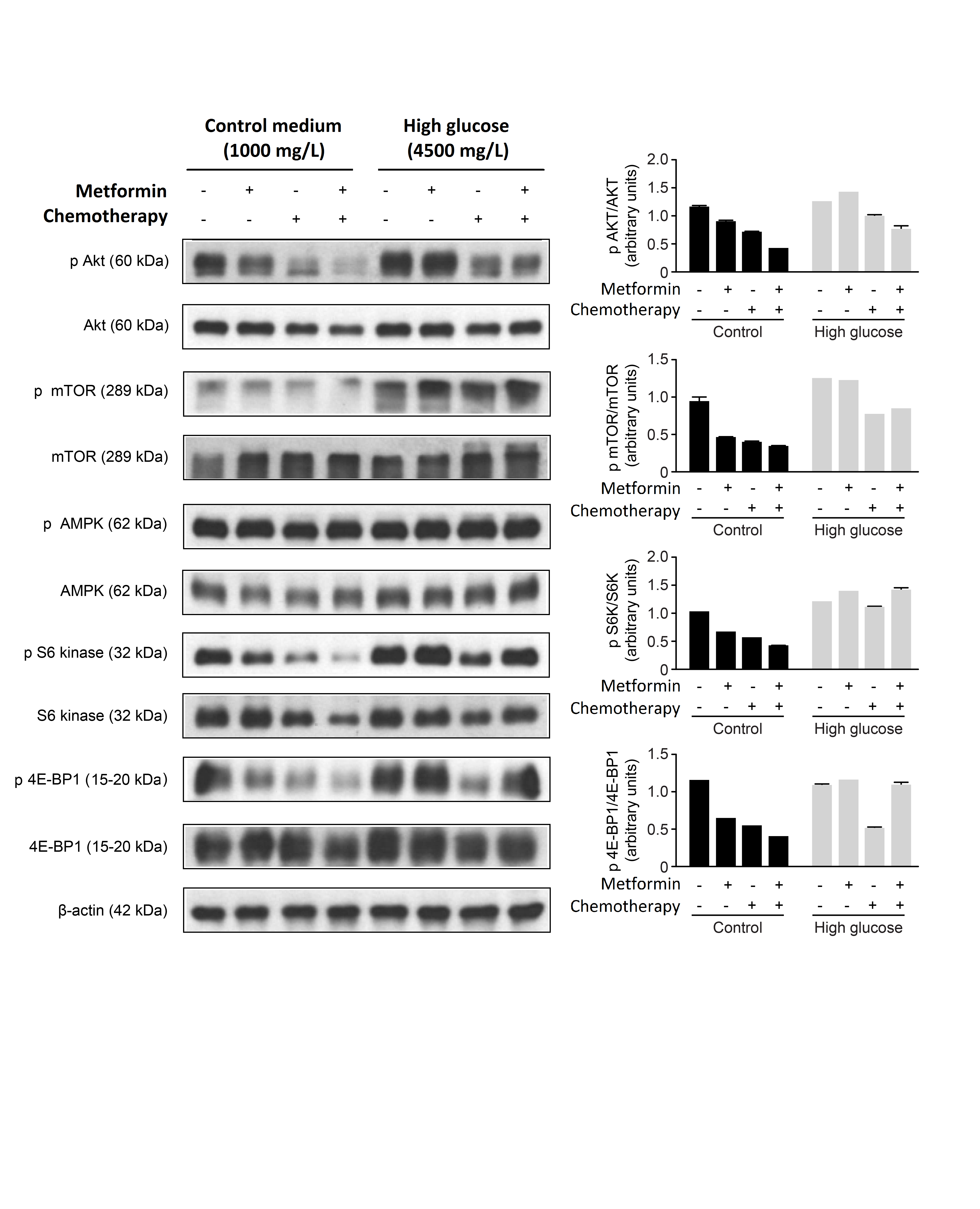

Supplement: Supplementary file 4 — Additional file 4: Figure S4. The effects of metformin in normal- or high-glucose culture medium. Western blotting was performed to assess the expression of AMPK, AKT, MTOR, 4E-BP1 and S6 following treatment with metformin, chemotherapy (carboplatin), or both for 48 h in the presence or absence of a high-glucose medium. Beta-actin was included as a loading control. [file 13048_2020_703_MOESM4_ESM.tif]
